# A New Remote Classroom Approach to Contouring Training for Pelvic Malignancies in Low- to Middle-Income Asian Countries

**DOI:** 10.7759/cureus.108054

**Published:** 2026-04-30

**Authors:** Yilan Liu, Caroline Carlson, Gregorius B Prajogi, Anuja Jhingran, Emma Holliday, Jonathan Paly, John Longo, Lindsay L Puckett, Nicholas G Zaorsky, Gustavo Sarria, Serguei A Castaneda, Benjamin C Li

**Affiliations:** 1 Radiation Oncology, University of Washington/Fred Hutchinson Cancer Center, Seattle, USA; 2 Radiation Oncology, Northwestern University Feinberg School of Medicine, Chicago, USA; 3 Radiation Oncology, Rumah Sakit Umum Pusat Nasional (RSUPN) Dr. Cipto Mangunkusumo Hospital, Jakarta, IDN; 4 Radiation Oncology, MD Anderson Cancer Center, Houston, USA; 5 Radiation Oncology, Massachusetts General Hospital, Boston, USA; 6 Radiation Oncology, Medical College of Wisconsin, Milwaukee, USA; 7 Radiation Oncology, University Hospitals Seidman Cancer Center, Case Western Reserve School of Medicine, Cleveland, USA; 8 Radiation Oncology, University of Bonn Medical Center, Bonn, DEU; 9 Radiation Oncology, CB Oncology, Cutler Bay, USA

**Keywords:** low- and middle-income country, online education, pelvic malignancies, radiation oncology education, radiation therapy contouring

## Abstract

Background and purpose: The feasibility, learner interest, and educational impact of online contouring training in low- and middle-income countries (LMICs) in Asia have not been documented on a large participant scale; hence, we aimed to investigate these factors while conducting a training program in contouring pelvic malignancies.

Materials and methods: A 10-session contouring course was offered to primarily radiation oncologists in Southeast Asia (SEA) via Zoom (Zoom Communications, Inc., San Jose, USA). The curriculum included case-based learning and supplemental reading materials focusing on common contouring scenarios and errors. Participation and pre- and post-program surveys, including demographics, confidence scores, a 10-question contouring knowledge test, and anonymous feedback, were recorded and analyzed. Changes in pre- and post-program scores were evaluated using a paired t-test.

Results: A total of 276 physicians participated in the curriculum, with 144 participants completing pre- and post-confidence score surveys and knowledge tests. The attendance for each session averaged 140 (range 115-158). Among paired participants, mean confidence scores increased from 2.92 to 3.67 out of 5 (+26.28%, p<0.01) and were significantly improved across all disease sites (range +21.5% to +34.1%). Mean knowledge test scores increased from 4.47 to 6.53 out of 10 (+46.2%, p<0.01). The average likelihood of recommending this training program to another colleague was 9.63 out of 10.

Conclusions: The pelvic malignancies contouring training program, which focused on avoiding common errors, resulted in robust participation, significant improvements in confidence and knowledge scores, and excellent course recommendation scores. This strategy could potentially be replicated for continuous medical education, scaled to other countries, and applied to other cancer disease sites in the future.

## Introduction

New efforts in radiation oncology are being developed to direct attention to underserved communities globally, increase the availability of effective treatments, and enhance knowledge sharing [1]. To date, several remote training courses tailored to radiotherapy teams and specific roles have demonstrated their feasibility and efficacy, including topics in high-dose rate brachytherapy for medical physicists, intensity modulated radiotherapy (IMRT) for radiation therapists, treatment planning of head and neck malignancies for radiation oncologists, and stereotactic body radiotherapy for multidisciplinary teams [2,3]. A gap remains in contouring training for target volume delineation, which is essential for delivering safe and effective radiation therapy [4,5]. Despite technical advances in radiation oncology, contouring consensus guidelines have proven to be difficult to follow and translate into routine clinical use [6,7].

Thus far, the feasibility, interest, and impact of remote, multi-session training programs in contouring for radiation oncologists in low- and middle-income countries (LMICs) in Southeast Asia (SEA) have not been widely reported. Existing initiatives have not been scaled for large numbers of participants, have not been conducted longitudinally over several months, have not been focused on this geographic region, and have not emphasized teaching about the common contouring errors. There are scarce reports on the methodology, efficacy, or scalability of educational contouring initiatives in LMICs that exist or have been published [3,8].

The goal of our study was to investigate the feasibility, interest, and impact of this approach in achieving broad geographic reach and high participation by utilizing an online training program designed for LMIC participation, with a focus on pelvic malignancies due to their regional and international prevalence. Furthermore, we uniquely focused on common contouring errors to explore their efficacy on learning outcomes. We hypothesized that this program could increase confidence and knowledge in target contouring for all pelvic malignancies. We hope that our methodology and findings will be useful for future courses in radiation oncology and planning scaling efforts in other LMICs.

This article was previously presented as a meeting abstract at the 2024 European Society for Radiotherapy and Oncology (ESTRO) Meets Asia Conference on August 24, 2024.

## Materials and methods

Participant recruitment 

Rayos Contra Cancer (RCC) partnered with the Southeast Asian Radiation Oncology Group (SEAROG) and Elekta Foundation to invite radiation oncologists from LMICs, particularly in SEA, to participate in contouring training via Zoom video conference (Zoom Communications, Inc., San Jose, USA). Radiation oncologists and senior radiation oncology residents were eligible to participate, as we recognized that even with established experience, there may have been limited contouring education during residency and/or outdated contouring practices that may need further refinement. Although this was designed for radiation oncologists in SEA, a few medical officers, medical physicists, and medical physics residents were allowed to join the study, as there were no specific exclusion criteria for participation, and the small numbers were unlikely to influence our aggregate study results. Each member country’s national radiotherapy society leadership was involved in disseminating the information through their local national membership networks. In total, 276 participants enrolled in the course. 

Curriculum

Eight radiation oncology faculties from the United States with sub-specialty expertise in pelvic malignancies were recruited to design and implement 10 live teaching sessions, each approximately 1.5 hours long, through Zoom video conference. The training format was similar to previous RCC programs [2,3,9,10]. Since most of our target audience already had some level of contouring training during residency, the curriculum was designed around avoiding common errors in contouring rather than focusing on the fundamentals. The topic of each session is described in Table 1.

**Table 1:** Pelvic malignancies contouring curriculum Post-op: Postoperative

Curriculum
Foundational skills of contouring
1. Recognizing your skills of contouring
2. Pelvic nodal contouring
Disease site-specific primary target contouring
3. Cervical cancer
4. Endometrial cancer
5. Prostate cancer - intact
6. Prostate cancer - post-op
7. Bladder cancer - intact
8. Rectal cancer
9. Anal cancer
10. Vulvar cancer

To design the curriculum, three senior authors developed an outline of common mistakes across different disease sites. The preliminary design was based on combined personal experience in education and published papers about common errors or inconsistencies in different disease sites [11,12]. We then conducted a broad needs-based assessment survey about contouring in SEA and seven semi-structured virtual interviews (43-84 minutes each) with radiation oncologists from different representative Southeast Asian countries working in various types of clinics to explore these needs, ensure our curriculum covered these, and to gain their feedback on our curriculum content and design. These virtual interviews were developed by the authors; an overview of the interview questions is presented in Supplement 1. The curriculum highlighted changes between three-dimensional conformal radiation therapy (3DCRT) contouring guides, which rely on bony anatomy, and IMRT contouring guides, which often use vascular and soft tissue anatomy, improving acute and chronic toxicity rates in organs at risk (OARs) and enhancing patients' quality of life [12]. Once finalized, the curriculum outline was shared with the volunteer educators, who were given the agency to follow the outline and add more learning points at their discretion. The detailed outline, including objectives for each teaching session, is presented in Supplement 2.

Educators focused on how to avoid common contouring errors and developed pictorial multiple-choice questions illustrating common mistakes, as shown in Supplement 3. Each knowledge-based question consisted of four visual options: one correct contour based on well-accepted international guidelines and three distractors showing common contouring mistakes. Prior to finalization and usage, these questions were trialed by four volunteers who provided live feedback during video calls. Educators also developed self-confidence questions specific to an individual’s ability to produce high-quality contouring, as shown in Supplement 3, such as “Please indicate how confident you currently are in your ability to contour EVERY target volume without ANY errors” (assessed for each different disease site), among others. The result was a curriculum tailored by experts to suit the common needs and interests of LMIC practicing radiation oncologists.

The sessions were conducted once a week between November 4, 2022, and January 28, 2023, in English. Live questions were answered with the support of session moderators trained via a standard onboarding procedure to facilitate an engaging and safe learning environment. Each session was recorded and made openly available online. Additionally, the course included supplemental materials such as pre-session readings and summary notes after each session.

Data analysis

The feasibility and learner interest in the training program were determined by recording and analyzing the number of attendees throughout each session, as well as qualitative feedback from participants. The educational impact of the training program was assessed by recording and analyzing the change in pre- and post-training confidence and knowledge scores.

Real-time attendance was recorded based on registration and self-reporting of attendance. The attendance rates were stratified between radiation oncologists and radiation oncology residents and plotted using Microsoft Excel (Microsoft Corp., Redmond, USA). A certificate of completion, indicating total hours of participation, was awarded to participants who completed the pre-course enrollment and post-course evaluation forms (described next) and attended at least one session.

The pre- and post- training surveys through Google Forms (Google LLC, Mountain View, USA) included participation consent, demographic data (including self-reported English proficiency), self-confidence scores using a 1-5 Likert scale across several domains, a knowledge test containing 10 multiple-choice questions as described above, and qualitative feedback [13]. The mean pre- and post-training confidence scores and knowledge scores for each participant were paired for analysis. The percent change in the respective scores and the p-values were determined using paired two-sample t-tests for means using Microsoft Excel. To handle multiplicity across many confidence domains, the mean confidence score and its p-value were reported as the composite endpoint.

Participants were asked to provide anonymous feedback on a Google Form that included a 1-10 rating of the course. “How likely are you to recommend an RCC program to someone considering participating?” was asked. Additionally, free-text response feedback was used to report qualitative data. These surveys were designed to measure pre- and post-knowledge or educational metrics to understand the participants' skills and demographics and to gather information to improve future curricula.

Ethics statement

This research was conducted in accordance with the Declaration of Helsinki. Informed consent was obtained, and all participant information was kept confidential and anonymized. Given the non-clinical nature of this study, it was exempt from institutional review board (IRB) approval.

## Results

Participants 

A total of 276 participants from 10 different LMICs registered for the course (Table 2). Of these, 181 participants were attending physicians, 90 participants were residents or trainees, and five participants had other roles in radiation oncology: two medical officers, two medical physicists, and one medical physics resident. These five participants did not have prior contouring experience. Most received three years (45.7%) or four years (29.7%) of radiation oncology-specific training as part of their residency. A quarter (24.6%) of participants had 0-2 years of contouring experience, a third (34.8%) had 3-5 years of contouring experience, and the rest (40.5%) had over five years of contouring experience. Most contoured 1-50 or 51-200 cases per year (46.7% and 34.4%, respectively), while a few contoured over 200 cases per year (18.8%). Most participants (90.6%) had 3D contouring taught during residency, while 26 participants (9.4%) did not. 

**Table 2:** Participants' demographic data *Two medical officers, two medical physicists, and one medical physics resident. **Inaccurate responses were recorded as “Other.”

Characteristics	Frequency (n=276)
Role in the radiotherapy department	
Attending	181 (65.6%)
Resident or trainee	90 (32.6%)
Other*	5 (1.8%)
Country of origin	
Indonesia	100 (36.2%)
Malaysia	72 (26.1%)
Myanmar	36 (13.0%)
Thailand	23 (8.3%)
Philippines	22 (8.0%)
Bangladesh	11 (4.0%)
Singapore	7 (2.5%)
Cambodia	2 (0.7%)
Papua New Guinea	2 (0.7%)
Pakistan	1 (0.4%)
3D contouring taught during the participant’s residency	
Yes	250 (90.6%)
No	26 (9.4%)
Years of residency training that were/are focused on radiation oncology	
0	1 (0.4%)
1	15 (5.4%)
2	30 (10.9%)
3	126 (45.7%)
4	82 (29.7%)
5	14 (5.1%)
6 or more	3 (1.1%)
Not applicable*	5 (1.8%)
Years of contouring experience	
0	10 (3.6%)
1	23 (8.3%)
2	35 (12.7%)
3-5	96 (34.8%)
5-9	74 (26.8%)
10-19	35 (12.7%)
20+	3 (1.1%)
Amount of patient contours performed yearly at each participant’s site	
0	3 (1.1%)
1-50	129 (46.7%)
51-200	95 (34.4%)
201-400	27 (9.8%)
401-600	10 (3.6%)
601-800	3 (1.1%)
801-1000	6 (2.2%)
1000+	1 (0.4%)
Other**	2 (0.7%)

Primary outcomes 

**Figure 1:**
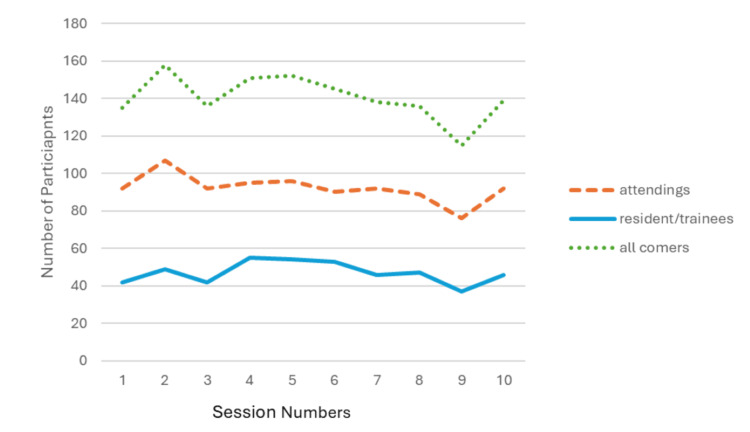
Synchronous online participation per session Trend of participation by attendings, resident/trainees, or all comers per session over time. See Table 1 for a description of each session. Note that “attendings” refers to post-radiation oncology residency physicians and “resident/trainees” refers to physicians currently in a radiation oncology residency training program.

**Figure 2:**
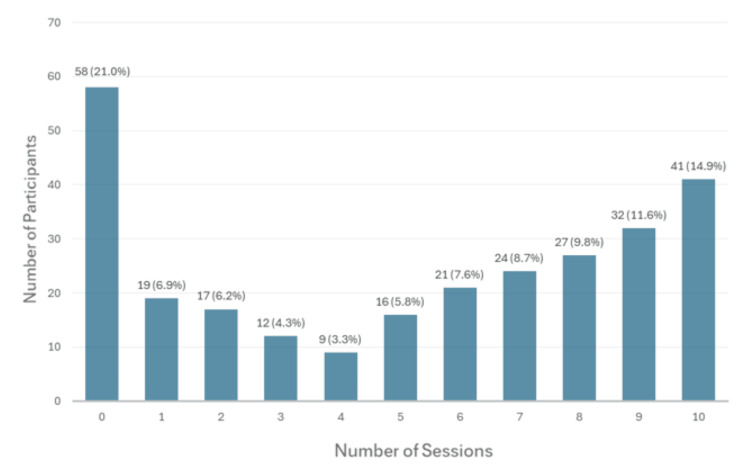
Number of sessions attended by all participants The number of sessions attended per participant is reported for 276 participants. For example, 21 participants attended six out of 10 live sessions and 41 participants attended 10 out of 10 live sessions.

Overall session attendance or participation was steady among attending physicians and resident trainees throughout the 10 sessions (Figure 1), with a mean of 140 participants per session (range 115-158). Per participant, the average number of sessions attended throughout the program was 5.1 (standard deviation 2.36); 41 participants attended all 10 sessions of this course (Figure 2). Out of 276 enrolled participants, 58 did not attend any live sessions.

**Figure 3:**
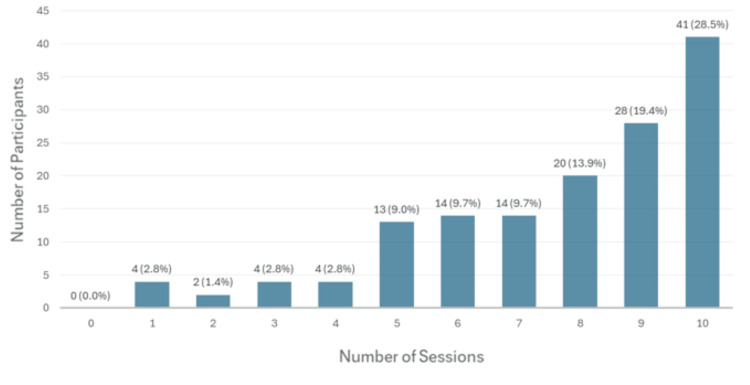
Number of sessions attended by paired participants The number of sessions attended per participant is reported for the 144 participants who submitted the pre- and post-training survey. For example, 20 participants attended eight out of 10 live sessions.

Afterwards, 144 out of 276 (52.2%) participants had pre- and post-training data that were included in the paired analysis. If we exclude the participants who signed up only for the course material and did not attend the sessions (n=58), 66% (144/218) of the participants completed the pre- and post-surveys. Among paired participants with pre- and post-survey scores, the average number of sessions attended was 7.7 (standard deviation 3.68) (Figure 3). Comparing pre- and post-program scores, significant improvements were seen across all 12 individual confidence domains spanning different disease sites, as shown in Table 3. The mean aggregate pre- and post-program confidence scores among 144 participants were 2.92/5 and 3.67/5, respectively (+26.28%, p<0.01). Similarly, as shown in Table 4, the mean pre- and post-program knowledge test scores among the 144 participants were 4.47/10 and 6.53/10, respectively (+46.19%, p<0.01).

**Table 3:** Pre- and post-program confidence scores The p-values were determined using the paired two-sample t-test for means. Post-op: Postoperative

Domains of confidence assessed	Pre-program mean confidence (1-5), all comers (n=276)		Pre-program mean confidence (1-5), completing participants (n=144)	Post-program mean confidence (1-5), completing participants (n=144)	Paired confidence change between pre- and post-completion of training	p-value
Ability to contour EVERY target volume without ANY errors for the following disease sites						
Cervical cancer (external beam contouring)	3.23	3.24		3.93	+21.5%	<0.01
Cervical cancer (brachytherapy contouring)	2.82	2.88		3.56	+23.9%	<0.01
Endometrial cancer	3.16	3.18		3.86	+21.4%	<0.01
Intact prostate cancer	3.01	2.97		3.71	+24.8%	<0.01
Post-op prostate cancer	2.75	2.71		3.53	+30.3%	<0.01
Bladder cancer (intact)	2.96	2.94		3.74	+27.2%	<0.01
Rectal cancer (intact)	3.18	3.18		3.88	+22.1%	<0.01
Rectal cancer (post-op)	2.95	2.92		3.66	+25.2%	<0.01
Anal cancer	2.75	2.69		3.54	+31.4%	<0.01
Vulvar cancer	2.58	2.60		3.49	+34.1%	<0.01
Ability to contour the pelvic lymph node regions without ANY errors, accounting for differences in how they are drawn between disease sites.	2.94		2.98	3.76	+26.3%	<0.01
Ability to teach skills involved in contouring to a junior team member	2.68		2.69	3.51	+30.4%	<0.01
Total average of all confidence domains	2.92		2.92	3.68	+26.3%	<0.01

**Table 4:** Pre- and post-program knowledge scores The p-values were determined using the paired two-sample t-test for means.

	Pre-program mean test score (out of 10), all comers (n=276)	Pre-program mean test score (out of 10), completing participants (n=144)	Post-program mean test score (out of 10), completing participants (n=144)	Paired test score change between pre- and post-completion of training (n=144)	p-value
Contouring knowledge	4.51	4.47	6.53	46.20%	<0.01

Additionally, 76 participants anonymously gave qualitative feedback. The mean satisfaction score was 9.63/10 (range 7-10). Participants reported satisfaction with the typical session length, time, and pace. Some representative responses were: “Very relevant course in our daily practice,” “It is very well structured, focused and meets all the demands of refining contouring skills. Educators are well versed. So, it has everything that a good course with great contents have in it,” and “I gained good knowledge and cleared doubts about misconceptions.”

## Discussion

This distinct training program, focusing on common errors in pelvic malignancies contouring, reached 276 participants across SEA and had a mean attendance of 140 participants per 1.5-hour session. Participants’ mean pre- vs. post-program confidence scores increased from 2.92 to 3.67 out of 5 (+26.28%, p<0.01), and knowledge test scores increased from 4.47 to 6.53 out of 10 (+46.19%, p<0.01). Participants demonstrated astute interest and engagement in topics covering common errors in contouring, which was further supported by a mean course recommendation score of 9.63 out of 10. Altogether, providing free, structured didactic sessions tailored to disease-specific contouring with an emphasis on common contouring mistakes was an effective curriculum design for radiation oncologists in LMICs.

Compared to prior education interventions on contouring, our team’s approach had unprecedented success in enrolling a large number of participants. One systematic review of educational interventions on contouring evaluated 16 out of 598 papers that had comparative data of pre- vs. post-educational intervention [14]. Of these 16 papers, seven involved pelvic malignancies, such as rectal and prostate cancer, and had a participant count in the tens range, while our training program was able to reach hundreds. While prior studies were not geared to train large numbers of clinicians in one setting, the training gap in LMICs calls for large-scale training initiatives [15-19]. We believe that the significantly high attendance and participation were measures of interest and satisfaction in the training program, indicating the success of our methods and curricular design.

In terms of factors that affect participation, the platform and teaching methods were important to consider. The aforementioned systematic review showed that the mode of teaching, whether onsite, online, or blended, did not impact the improvement in contouring among learners [14]. To this point, a study on IMRT contouring training for 37 participants from three centers in LMICs evaluated the effectiveness of different types of virtual teachings: theoretical, hands-on, or self-guided [8]. Using a survey design, they showed that virtual hands-on (case-based) training was most helpful for participants, followed by virtual theoretical education sessions [8]. In our program, we were similarly comfortable with purely virtual training, as this improved accessibility and allowed hundreds of participants to join from 10 countries. Our teaching intervention combined synchronous (theoretical, interactive, and case-based learning with examples that reinforced teaching points) and asynchronous (course folders with educational materials and pre-readings) components that facilitated excellent large-group participation. Although 58 of the 276 enrolled participants attended 0 sessions, some participants enrolled with the intention of watching recorded sessions and accessing course materials (even if they had no plans to attend the live sessions). It is worth noting that these are different forms of engagement from the participants and that live participation is not a comprehensive proxy for overall engagement and interest in the course. Our findings support those reported in previous studies while also introducing a colorful approach that blends multiple virtual synchronous and asynchronous components at once to cater to very large groups of learners at a time.

Other studies have also referenced protocols and contour guidelines, combining these with quantitative impact assessments [20,21]. They have shown that experimental groups with access to the atlas or protocol had more uniform contouring across participants and achieved better overlap with the atlas or protocol volumes and dosimetric impacts, resulting in improved target coverage [20,21]. Our study design did not measure participant contours, as it was designed to assess a much larger number of participants. With more resources, contouring assessments could be conducted on a large scale, and we would have reason to expect a similar impact with the use of similar resources, combined with additional synchronous educational elements.

Finally, we designed specific curriculum objectives based on addressing common errors in target contouring, which, to our knowledge, were novel and filled a niche gap in education globally. In particular, we engaged educators and learners with pictorial multiple-choice questions throughout each session with four options: one answer as the key (showing a correct contour based on well-accepted international guidelines) and the other three as distractors (showing common contouring mistakes). The curriculum went beyond simply asking participants to follow a guideline or protocol; it had them clarify mistakes, targeting a higher level of learning. This approach was rated quite positively. In fact, the only disappointment expressed by participants regarding the content stemmed from the introduction session being too “introductory” or providing nothing new, revealing that the audience desired new and useful information rather than a repeat of the basics of gross tumor volume (GTV), clinical target volume (CTV), etc. Thus, there may be a large opportunity for filling the radiotherapy educational gap in “Level 2” technical training skills: beyond the basics but still accessible to broad audiences in LMICs, such as those in SEA.

Our study had several limitations. First, it was conducted in English, which was a second language for most learners. This could have limited the acquisition and application of the knowledge. Relatedly, the assessment tools used to report the training's impact may not fully convey the program's efficacy. Self-reported confidence scores have inherent biases, such as expectation bias, hindsight bias, and self-report bias. Our knowledge tests were only 10 questions long (representing each of the topics in the 10-session training program) and served as a signal rather than a comprehensive assessment of knowledge. A longer pre- and post-knowledge test could be considered, while respecting the time burden on participants completing these assessments. Furthermore, although the knowledge questions assess the participant’s ability to identify the correct contours, the study did not explicitly evaluate participants’ individual contours; thus, we cannot prove that their contouring skills have definitely improved and translated into clinical practice. Additionally, there was potential for selection bias toward more engaged participants in the paired analysis and limitations inherent to attendance metrics based on self-report rather than objective platform logs. There was attrition bias as only 52.2% of participants completed paired pre- and post-surveys. If we exclude the participants who signed up only for the course material and did not attend the sessions (n=58), 66% (144/218) of the participants completed the pre- and post-surveys. We acknowledge that the multiplicity across many confidence domains in our survey may have introduced a higher risk of false positives in the study. Qualitative analysis and non-response bias were additional limitations.

Traditional models of impact assessment have often emphasized patient-level outcomes; however, such approaches are frequently costly and difficult to implement in LMIC settings. Although our study methodology cannot serve as a benchmark for evaluating clinical impact, it reflects an educational innovation report, reaching hundreds of clinicians across multiple countries simultaneously. This program builds on earlier educational initiatives while raising the bar for what can be achieved at scale. We propose this approach as an emerging paradigm for measuring the success of global oncology training, one that can be further strengthened by correlating educational outcomes with patient outcomes at selected pilot centers.

## Conclusions

Our 10-session weekly remote contouring training program focused on avoiding common errors resulted in consistent participation, strong course recommendation scores, and significant improvements in confidence and knowledge. Providing structured didactic sessions on disease-specific contouring with combined synchronous and asynchronous educational components was a highly effective curriculum design for radiation oncology attending physicians and residents in LMICs in SEA. The success of this initiative demonstrates the feasibility of implementing a scalable, resource‑efficient training model and highlights the program as a meaningful educational innovation that enhances learner outcomes. This strategy could be replicated for continuous medical education, scaled to other countries, and applied to other cancer disease sites in the future.

## Figures and Tables

**Figure 4:**
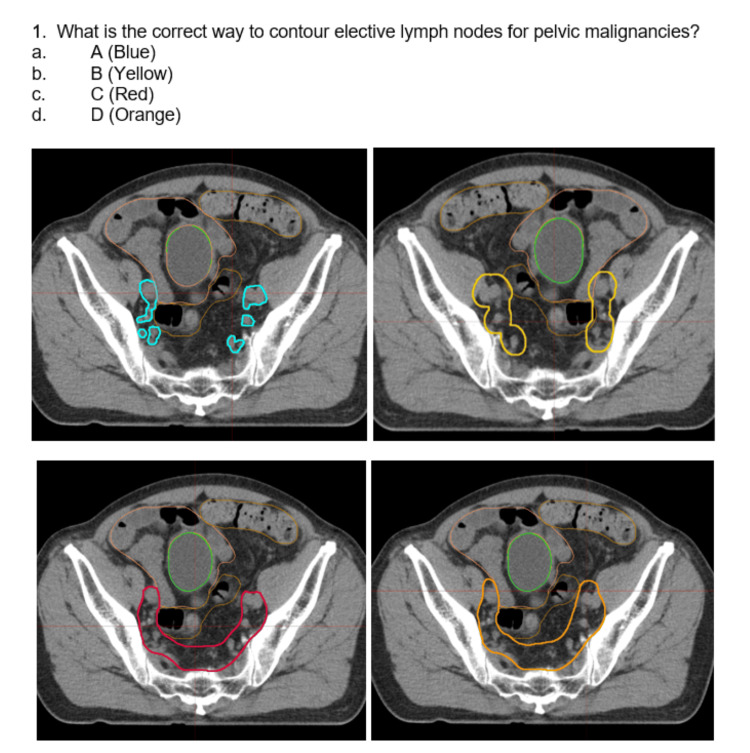
Question 1 Image used with permission from Rayos Contra Cancer.

**Figure 5:**
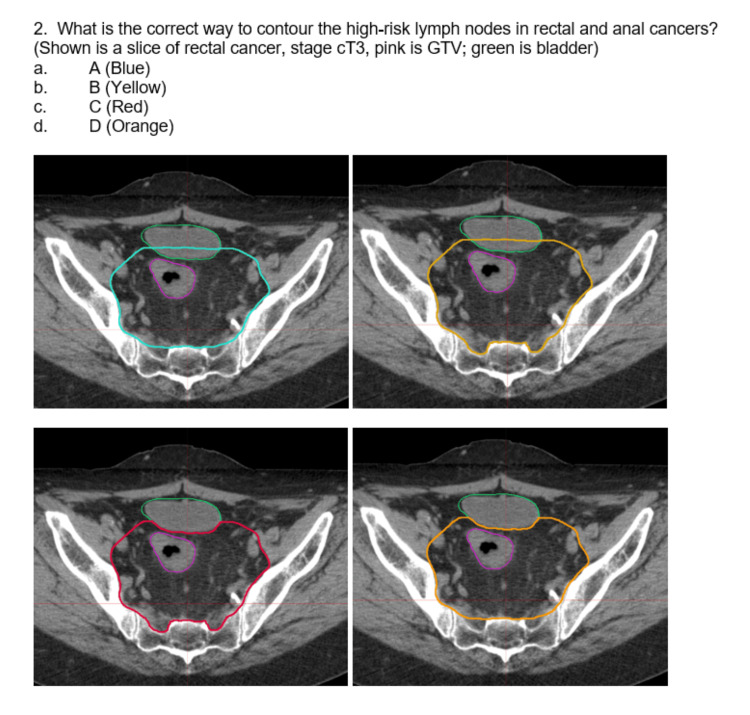
Question 2 Image used with permission from Rayos Contra Cancer. GTV: Gross Tumor Volume

**Figure 6:**
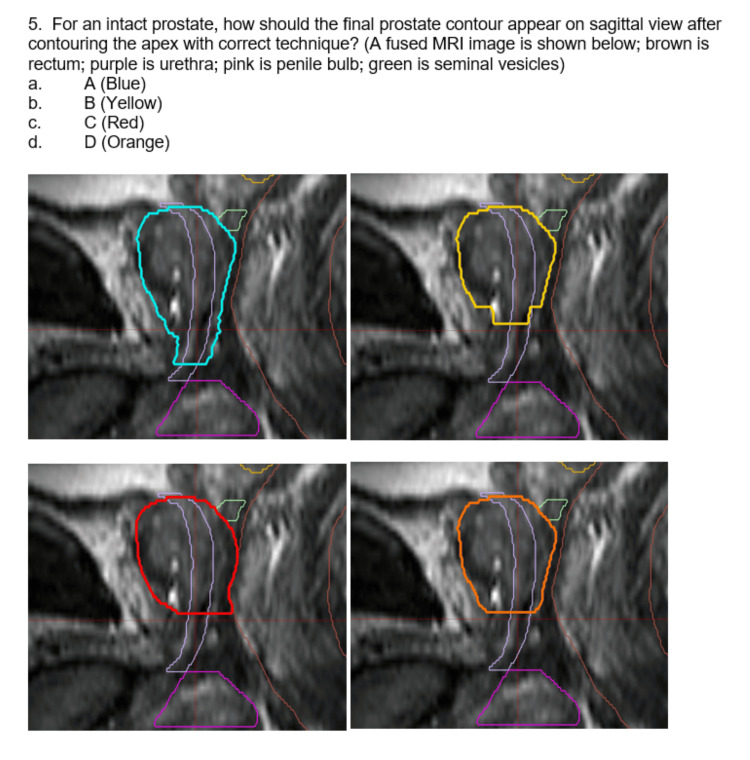
Question 5 Image used with permission from Rayos Contra Cancer.

**Figure 7:**
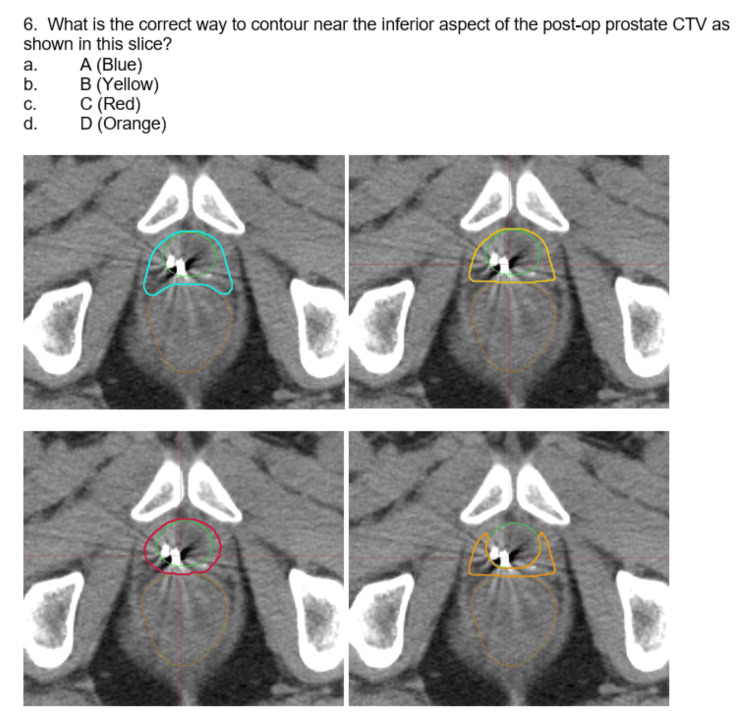
Question 6 Image used with permission from Rayos Contra Cancer. CTV: Clinical Target Volume; Post-op: Postoperative

**Figure 8:**
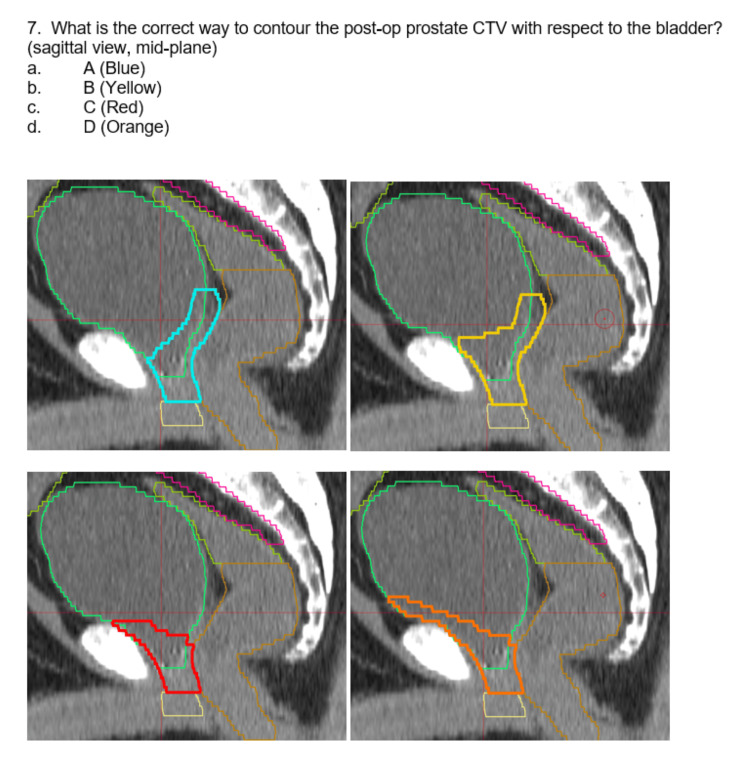
Question 7 Image used with permission from Rayos Contra Cancer. CTV: Clinical Target Volume; Post-op: Postoperative

**Figure 9:**
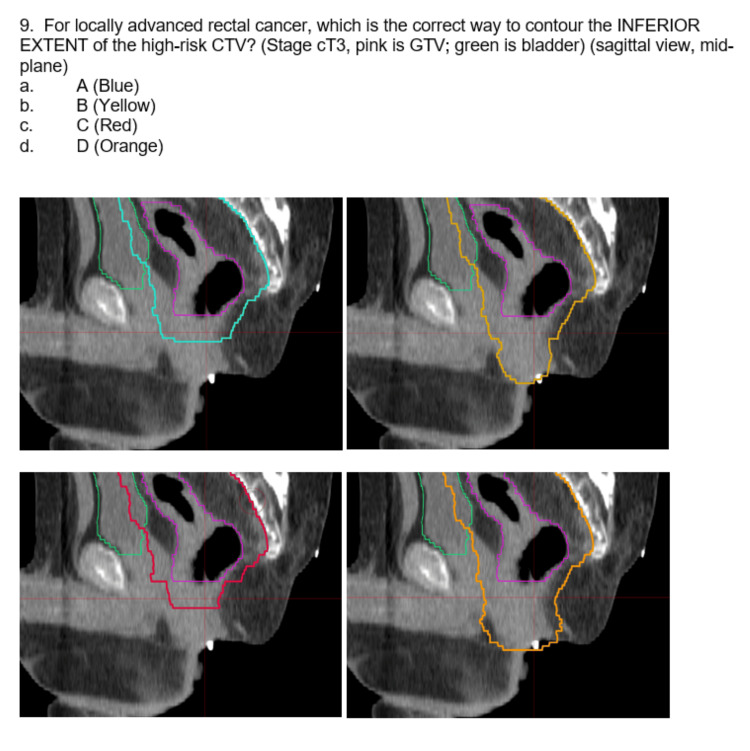
Question 9 Image used with permission from Rayos Contra Cancer. CTV: Clinical Target Volume; GTV: Gross Tumor Volume

**Figure 10:**
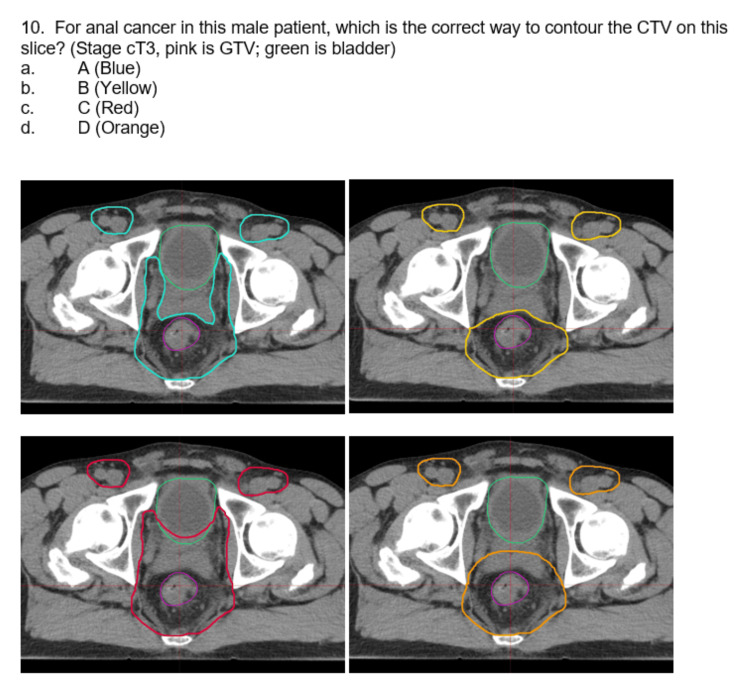
Question 10 Image used with permission from Rayos Contra Cancer. CTV: Clinical Target Volume; GTV: Gross Tumor Volume

## Appendices

Supplement 1: Semi-structured virtual interview

**Table 5:** Semi-structured virtual interview overview All of the above questions were provided as a suggested framework to guide interviewers. However, interviewers were encouraged to adapt the wording and sequence of questions as needed to maintain a natural and productive flow of conversation. These questions were used for curriculum planning and assessment of the planned curriculum, confidence questions, and knowledge questions. RCC: Rayos Contra Cancer; IMRT: Intensity Modulated Radiotherapy; 3DCRT: Three-Dimensional Conformal Radiation Therapy; EST: Eastern Standard Time

Suggested framework	Details
Introduce yourself	Example: “My name is [X], I am from RCC. Thank you for agreeing to help us with this new contouring course called How to Avoid Common Errors in Contouring Pelvis Cancers. It will be 9 weeks long and start in November.”
Ask for permission to record	Confirm and ensure the call is recording.
Clinic information	Ask: “What clinic do you work for?”
Confirm specialty	Verify they are a radiation oncologist.
Personal experience	Ask: “How long have you been a radiation oncologist?”
Training	Ask: “What is the training or residency like in your country?”
Clinic workflow	Ask: “What is it like to practice in your country day to day?”
Treatment planning	Ask: “What is the breakdown of IMRT vs. 3DCRT use?”
Disease site specifics	Ask: “What are the needs per specific disease site in terms of contouring training and/or treatment?”
General	Ask: “What else would you like to share that may be helpful to include in the training program?”
Course timing preference	Ask: “Which option would be preferred?” Option 1: Saturday Evening - 7:30 pm Bangladesh/8:00 pm Myanmar/8:30 pm Indonesia & Thailand/9:30 pm Philippines & Malaysia/Saturday 8:30 am EST. Option 2: Sunday Morning - 7:00 am Bangladesh/7:30 am Myanmar/8:00 am Indonesia & Thailand/9:00 am Philippines & Malaysia/Saturday 8:00 pm EST.
Interest	“Given how I’ve described the course, is it interesting to you? Is it too hard, too basic, or just right?”
Content	After reading through the details of the Curriculum Syllabus (Supplement 2), ask: “What are your thoughts or reactions to this?”
Confidence questions	“Please read out loud each question and let us know if there is anything about the question that is confusing or notable.”
Knowledge questions	“Please read out loud each question and let us know if there is anything about the question that is confusing or notable. What do you think the answer is?” Share the answer and explanation. “Please tell me what you think of the answer/explanation and how difficult the question was to you.”

Supplement 2: Contouring curriculum outline

This contouring curriculum covers 22 common scenarios and is divided into three parts: Pelvis (Sessions 1-10), Head (Sessions 11-18), and Thorax/Abdomen (Sessions 19-23). Each part is considered a distinct program that participants can enroll in. Each program consists of once-per-week sessions, each lasting 1-1.5 hours. Sessions will be held on Zoom, and attendance will be recorded and credited toward certificates. There is no cost associated with the program.

The skills emphasized in this curriculum include distinguishing gross disease on imaging, using additional available medical information efficiently, contouring anatomic volumes and expansions, and applying contouring tools, resources, and mental tricks.

The target audience is radiation oncologists with IMRT and prior contouring experience. The teaching approach should be a mix of didactic methods (such as lecture slides) and practical demonstrations (such as showing live cases with contouring errors using a treatment planning system (TPS) platform). The ideal approach is to share slides that explain concepts, then switch to RCC ProKnow (Elekta AB, Stockholm, Sweden) to show examples and talk through them, including the thought process when making revisions and highlighting common errors. It is recommended to show cases with messy, real-life anatomy.

Educators should keep in mind several tips: first, show what a good example looks like. Then, show common errors by using a separate structure set already built (for example, “CTV with errors”), examine it, critique it, and demonstrate corrections. Be familiar with the contours of your good example to avoid surprises that could confuse learners. When demonstrating errors, it is better to show existing errors and how to correct them rather than intentionally drawing incorrect contours, which can be disorienting. To improve visibility, increase contour line thickness, and delete any irrelevant contours such as skin, body, couch, or planning structures.

Supplemental tools for learning include sharing pre-reading material for anatomy review before sessions and incorporating Zoom poll questions into lecture slides. Examples include: “How often do you contour X kinds of cases?” or “Which is the correct way to contour this slice?” with four options. Moderators will also prepare a summary sheet for each lecture listing main points and common errors, which will be shared with educators for approval afterward.

**Table 6:** Recognizing your skills of contouring (Session 1) H&N: Head and Neck; GI: Gastrointestinal; GYN: Gynecologic; GU: Genitourinary; ENE: Extranodal Extension; LVI: Lymphovascular Invasion; PNI: Perineural Invasion; ITV: Internal Target Volume; PTV: Planning Target Volume; TPS: Treatment Planning System; GTV: Gross Tumor Volume; RCC: Rayos Contra Cancer; Post-op: Postoperative; Pre-op: Preoperative; Sim: Simulation

Section		Details
Framework and skills	Overview	This course provides a framework for you to self-evaluate and improve your contouring skills as a radiation oncologist.
	Skills focus	- Distinguishing gross disease on imaging. - Using additional available medical information efficiently. - Contouring anatomic volumes and expansions. - Applying contouring tools, resources, and mental tricks.
Distinguishing gross disease on imaging	Image registration	How to apply image registration (also called “image fusion”) of MRI or PET/CT: Don’t look side by side to estimate where the gross disease is, because this method is error-prone. Learn to import prior imaging, perform image registration, adjust as needed so it is accurate, and look at fusion to be exact!
	CT Sim with IV contrast	When both advantageous and available at your institution, do CT Sim with IV contrast: H&N, lung, GI, definitive GYN, sarcoma, lymphoma. CT contrast not useful for CNS (MRI preferred), most GU, skin, post-op GYN/GI.
	Other discussion points	- Discuss the GTV definition. - Sometimes best to do normal structures before targets (explanation for why). - How to use window levels to help distinguish difficult anatomy. - Don’t forget to try changing the window level if you have a difficult time delineating the extent of disease/anatomic borders. - View contoured gross disease on sagittal/coronal imaging as a double check. - Choose an anatomic plane/boundary, and stick with it; don’t jump around.
Using additional available information	Surgical and endoscopic reports	- Don’t forget to see if the surgeon struggled to clear the tumor in any particular areas. - Tumors in luminal structures (pharynx, rectum, anus, esophagus) are described in reports. Look for descriptions of the distance and extent of the tumor.
	Pathology reports	Review margins, nodal involvement, ENE/LVI/PNI.
	Physical exam	- Perform direct visualization or palpation when possible. - Look for subtle signs of nerve involvement.
	Prior imaging	- Three ways: glance, eye-ball (bad), register (good). - Handling different positions: focus fusion on the area of greatest concern, create multiple fusions if needed. - Which prior imaging to fuse: pre-op, post-op, post-neoadjuvant.
Contouring anatomic volumes and expansions	CTV	- Definition. - Correctly applying expansions (size, respecting boundaries). - Post-op volumes (tumor bed vs. standardized region). - Elective volumes (include/exclude nodal basins, nerve coverage). - ITVs (motion-based, not covered). - PTV expansions (definition, origin). - Handling uncertainty. - Keeping planner in mind.
Contouring tools and mental tricks	Hotkeys	Brush/Pen, Smooth, Interpolate, Fill, Clear, Zoom in/out, Pan, Toggle fusion views.
	TPS setup	Eclipse (Varian Medical Systems, Palo Alto, USA), Monaco (Elekta AB, Stockholm, Sweden), MIM (MIM Software, Inc., Cleveland, USA), RayStation (RaySearch Laboratories, Stockholm, Sweden), Other.
	Resources	Consensus guidelines, organized references.
	Educator narration	Mental tricks demonstrated during live cases.
General	Pre-readings	Flipped classroom approach.
	Resources list	eContour.org [22], RadOncReview [23], Radiopaedia [24], e-Anatomy [25], RCC videos [26].
	Goal	By the end of this course, we hope you will be empowered to contour efficiently while avoiding common errors!

**Table 7:** Pelvic nodal contouring (Session 2) LN: Lymph Node; SCC: Squamous Cell Carcinoma; EBRT: External Beam Radiation Therapy; CBCT: Cone Beam Computed Tomography; Sim: Simulation; GYN: Gynecologic; ITV: Internal Target Volume; CTV: Clinical Target Volume; 3DCRT: Three-Dimensional Conformal Radiation Therapy; GI: Gastrointestinal; IMRT: Intensity Modulated Radiotherapy; SIB: Simultaneous Integrated Boost; Post-op: Postoperative

Section	Details
Pre-reading	- eContour example cases [22]. - Pelvic LNs post-op GYN updated NRG guidelines - Small et al. 2021 [12]. - Prostate LNs contouring guidelines - De Hertogh et al. 2023 [27]. - Prostate LNs guidelines NRG updated - Hall et al. 2021 [11].
Anatomic landmarks and common mistakes for key nodal stations	Common iliacs, internal iliacs, external iliacs, presacral, obturator, inguinal, para-aortic: - Superior/inferior extent landmarks. - Show small bowel vs. large bowel (these are commonly confused). - Highlight common muscles mistaken for bowel/vessels. - Cover space in front of sacrum (common mistake is to not contour into the crevice).
Appropriate expansions around vessels	- Should be 7 mm expansion. - Can be 10 mm expansion anterolaterally for external iliacs as they travel anteriorly to account for the gap between CT slices. Common mistakes: 1. Cover veins, not just arteries. 2. Don’t miss the branching vessels from the internal iliacs. They are subtle, but you should include them. 3. Width of tissue strip linking the external and internal iliac regions and encompassing the obturator region (different by disease site?).
Disease-specific nuances	GYN - Cervix: Are there any deviations from standard nodal contouring? i. Upper border of CTV: Don’t under- or over-contour the superior border. Default is to include the common iliacs up to where they join together = “large pelvis.” For low-risk patients (tumor <4 cm, N0, IA/IBI/IIA1, SCC, and no uterine invasion), the EBRT field may exclude the common iliacs and only go up to where the external and internal iliac arteries join = “small pelvis.” For high-risk patients, cover large pelvis + para-aortic LNs. Don’t confuse the indications, and don’t forget that the contouring depends on whether the para-aortics themselves are involved or not. Any common iliac node involvement or higher or ≥3 pelvic nodes. Nodes count if PET+ and ≥0.5 cm size with suspicious shape/high avidity, or ≥1.0 cm on short axis. If no para-aortic nodes are involved, the superior border is above the renal veins. If para-aortic nodes ARE involved, the superior border goes up to 3 cm above the highest involvement, or to renal veins, whichever is higher. ii. (Intact cervix) Presacral nodes down to S2/S3 vs. bottom of the sacrum. This is a nuanced detail with different practices in different centers. The main point is you don’t want to have recurrence. Are you ensuring consistent rectal filling everyday with CBCT? Are you including an ITV where you are confidently covering the anterior mesorectum? If you are in a high-resourced center (with MRIs, spending lots of time on planning, ITVs, and fastidious CBCT alignment), with confidence you aren’t having failures, then you can keep doing S2/S3; otherwise, the safe way to go is to cover the whole sacrum. There is minimal added rectal toxicity, and it prevents failures. iii. (Post-op cervix) Presacral nodes down to S2/S3 interspace.
	GYN - Endometrial: Are there any deviations from standard nodal contouring? Presacrals drawn the same as post-op cervix.
	Prostate: Prostate - are there any deviations from standard nodal contouring? i. Upper border of CTV: Don’t under- or over-contour the superior border. Default is to include the common iliacs up to where they join together at the bifurcation of the aorta. If extensive superior nodal involvement, then include the para-aortic nodes by going up to the first slice above all the renal veins. ii. Presacral nodes down to bottom of S3. Don’t stop at bottom of S2. Don’t need to cover the neural foramen because it’s uncommon for prostate cancer to fail there. iii. Where to end the external iliac nodes inferiorly… (taper off external iliac contours as the vessels enter the inguinal canal). How low to go? Keep contouring down until you see the medial edge of the external iliac arteries crossing past the medial aspect of the iliac bones. iv. Where to end the obturator nodes inferiorly… (when seminal vesicles enter prostate? Or when the obturator artery passes through the obturator foramen?).
	Bladder: Are there any deviations from standard nodal contouring?
	Rectal: Are there any deviations from standard nodal contouring? i. When drawing presacral nodes, you need to cover the neural foramen because this is a common area of rectal cancer recurrence ii. Do not crop out the bowel from the pelvic LN CTV contours. Compared to other cancer types, GI patients are at a higher risk of failure if you do this.
	Anus: Are there any deviations from standard nodal contouring? i. When drawing presacral nodes, you need to cover the neural foramen because this is a common area of anal cancer recurrence ii. Do not crop out the bowel from the pelvic LN CTV contours. Compared to other cancer types, GI patients are at a higher risk of failure if you do this.
Technique-based emphasis	Emphasize the difference between 3DCRT (bony anatomy guidelines) and modern IMRT guidelines. For modern 3D contouring, IMRT guidelines should be used. GYN/prostate/rectal/anal: L4/5 vs. bifurcation of common iliac arteries GYN/prostate/rectal/anal: including anterior ½ of vertebral body vs. excluding bone in CTV. Prostate: external iliac stopping at top of the acetabulum vs. after passing through the inguinal ligament, etc. All: treating pelvis without thinking about the need for SIB vs. looking carefully at CT Sim scan for any new suspicious LNs, especially if there is significant time between staging scan and CT Sim.

**Table 8:** Cervix (external beam and brachytherapy contouring) (Session 3) Onc: Oncology; HDR: High-Dose Rate; PTV: Planning Target Volume; CTV: Clinical Target Volume; OAR: Organ at Risk; LN: Lymph Node

Section	Details
Pre-session preparation	1. Watch Dr. Anuja Jhingran demonstrate external beam contouring https://www.youtube.com/watch?v=eo-gIbJx5Ck (3:37 - 47:48) [28]. 2. Watch Dr. Anuja Jhingran demonstrate brachytherapy contouring (example 1) https://www.youtube.com/watch?v=rgpsfJslSZQ [29]. 3. Watch Dr. Anuja Jhingran demonstrate brachytherapy contouring (example 2) https://www.youtube.com/watch?v=XrUjTPteEHo [30].
Pre-readings	- Cervical cancer consensus contouring guidelines IMRT - Lim et al. 2011 [31]. - EMBRACE II protocol [32]. - GYN radiation oncology practical guide - Chapter 5 contouring - Eifel and Klopp 2017 [33]. - Duodenal constraints - Lakomy et al. 2022 [34]. - HDR brachytherapy dose calculations Excel sheet from NRG [35].
Distinguishing gross disease on imaging	General tips.
Using additional available medical information efficiently	a. Physical exam. b. Prior imaging: - PET/CT - don’t neglect to review, register, and fuse to identify LN involvement that should be contoured for boosting to a higher dose. - MRI - don’t neglect to review to identify the extent of gross disease for brachytherapy planning and contouring.
Applying contouring tools, resources, and mental tricks	Educators demonstrate their style for how they contour and speak aloud their thoughts as they show contouring.
Contouring anatomic volumes and expansions	a. Bladder filling is challenging. b. Uterus CTV -> PTV expansion, uterus can move up to 3 cm per day. c. Contouring the vagina CTV. Guidance to make sure you don’t contour too far distally (vulvar toxicity). - Common misconception: “Oh, I should contour down to the introitus to prevent recurrence.” Reason this is wrong: overall, you are adding too much toxicity relative to the benefit. We have brachytherapy in the case of recurrence. d. OAR: bone marrow.

**Table 9:** Endometrium (Session 4) LN: Lymph node; GYN: Gynecology; Post-op: Postoperative

Section	Details
Pre-reading	Pelvic LNs post-op GYN updated NRG guidelines - Small et al. 2021 [12].
Distinguishing gross disease on imaging	General tips.
Using additional available medical information efficiently	General tips.
Applying contouring tools, resources, and mental tricks	Educators demonstrate their style for how they contour and speak aloud their thoughts as they show contouring.
Contouring anatomic volumes and expansions	a. Post-op endometrial - Don’t forget to do post-op imaging. Imaging is really important. - Don’t forget to consider when to do an extended field vs. not an extended field. Describe how to make this distinction. b. Inoperable endometrial.

**Table 10:** Intact prostate (Session 5) SV: Seminal Vesicles; IGRT: Image-Guided Radiation Therapy; SBRT: Stereotactic Body Radiation Therapy; Sim: Simulation; LN: Lymph Node

Section	Details
Pre-session preparation	Watch Dr. John Longo demonstrate external beam contouring https://www.youtube.com/watch?v=eo-gIbJx5Ck (1:48:00 - 2:16:06) [28].
Pre-readings	- eContour module: intact prostate [36]. - Normal male pelvis anatomy - Gay et al. 2012 [37]. - Prostate contouring errors - McLaughlin et al. 2019 [38]. - Prostate functional anatomy - McLaughlin et al. 2005 [39].
Distinguishing gross disease on imaging	a. Don’t forget there are new things you need to do for SBRT that you don’t need to do for conventional fractionation. - Don’t forget to place fiducial markers before CT Sim (to be able to track with IGRT). b. Applying correct MRI/CT registration. - Don’t click autoregister and then not check to see if adjustments are needed. - Don’t align to the bony anatomy. Align the fiducial markers from the MRI to the fiducial markers from the CT Sim.
Using additional available medical information efficiently	a. Medical record: You should know the risk-stratification of your patient (low, favorable-intermediate, unfavorable-intermediate, or high-risk) as this determines your contouring decisions. - Considering elective LNs (acknowledging this has been a debate; nonetheless, the radiation oncologist should use patient information to guide this decision). - Considering if and how much of the proximal seminal vesicles to include. b. Pathology report: Don’t forget to look if there is SV involvement. If SVs are involved, then the entire SVs should be included in CTV.
Applying contouring tools, resources, and mental tricks	Educators demonstrate their style for how they contour and speak aloud their thoughts as they show contouring.
Contouring anatomic volumes and expansions	a. Contouring the prostate apex. b. Contouring the prostate base. c. Contouring the penile bulb common errors: - Contouring the penile bulb anterior to the urethra or symphysis pubis. - Too small in diameter (like the size of the urethra). - Not as a round structure but as a large triangular, trapezoidal, or rectangular shape. - Too anteriorly. - Too superior and close to the prostate apex. d. Contouring the prostate urethra (with MRI for SBRT).

**Table 11:** Postoperative prostate (Session 6) VUA: Vesicourethral Anastomosis; CTV: Clinical Target Volume; ARRO: Association of Residents in Radiation Oncology; ESTRO: European Society for Radiotherapy and Oncology; ACROP: Advisory Committee for Radiation Oncology Practice; Post-op: Postoperative

Section	Details
Pre-session preparation	Watch Dr. John Longo demonstrate external beam contouring https://www.youtube.com/watch?v=eo-gIbJx5Ck (1:48:00 - 2:16:06) [28].
Pre-readings	- Prostate LNs guideline NRG updated - Hall et al. 2021 [11]. - Post-op prostate - Michalski et al. 2010 [40]. - Post-op prostate - Thompson et al. 2013 [41]. - Post-op prostate contouring ESTRO ACROP - Dal Pra et al. 2023 [42]. - Post-op prostate case ARRO [43]. - MedPortal: Gunther et al. post-op prostate and seminal vesicle fossae contouring module [44].
Distinguishing gross disease on imaging	(Doesn’t apply in this session).
Using additional available medical information efficiently	General tips.
Applying contouring tools, resources, and mental tricks	Educators demonstrate their style for how they contour and speak aloud their thoughts as they show contouring.
Contouring anatomic volumes and expansions	a. Don’t be shy about contouring just anterior to the rectum. This is a common area of recurrence. b. Don’t guess the inferior aspect of the post-op CTV. Identify the VUA and contour down to 8-12 mm below it. The VUA can be approximated as the last slice where urine is visible. - Can include more if concerned about the apical margin. c. Don’t guess the superior aspect of the post-op CTV. Identify the superior surgical field where there could have been seeding, which is marked by the cut end of the vas deferens. Cover surgical clips and seminal vesicle remnants.

**Table 12:** Intact bladder (Session 7) IJROBP: International Journal of Radiation Oncology, Biology, Physics; BCON: Bladder Carbogen Nicotinamide; ACR: American College of Radiology; NEJM: New England Journal of Medicine; BC2001: Bladder Cancer 2001; CTV: Clinical Target Volume; Hypofrac: Hypofractionated; Onc: Oncology

Section	Details
Pre-readings	- IJROBP bladder failure patterns - Baumann et al. 2013 [45]. - IJROBP development validation of bladder contouring atlas - Baumann et al. 2016 [46]. - Lancet Onc BC2001 BCON hypofrac meta-analysis - Choudhury et al. 2021 [47]. - IJROBP ACR bladder preservation guidelines - Dinh et al. 2019 [48]. - NEJM BC2001 bladder preservation - James et al. 2012 [49]. - We will focus just on the BC2001 protocol [49]. It’s unlikely in limited-resource settings that an intensive approach with cystoscopy would be chosen. Furthermore, newer data suggest that 55 Gy in 20 fractions (hypofractionation) should be adopted as a standard of care for bladder preservation [47]. - Radiotherapy guide - BC2001 supplement protocol [49].
Distinguishing gross disease on imaging	Contour the whole bladder, but also look for any tumor extending beyond the bladder wall in locally advanced cases.
Using additional available medical information efficiently	Don’t forget to rule out prostate cancer before treating the bladder. Otherwise, you may undertreat their prostate cancer and then be in a hard situation for re-irradiation.
Applying contouring tools, resources, and mental tricks	Educators demonstrate their style for how they contour and speak aloud their thoughts as they show contouring.
Contouring anatomic volumes and expansions	a. Maybe cover common questions about the prostate as a CTV? - How/when do you contour the prostate CTV? (Is it different from contouring the prostate in prostate cancer?)

**Table 13:** Intact and postoperative rectum (Session 8) RTOG: Radiation Therapy Oncology Group; CTV: Clinical Target Volume; GTV: Gross Tumor Volume; GYN: Gynecology; Chemo: Chemotherapy

Section	Details
Pre-readings	- International consensus guidelines on CTV delineation in rectal cancer - Valentini et al. 2016 [50]. - Anorectal contouring atlas for RTOG - Myerson et al. 2009 [51]. - eContour module: rectal cancer [22].
Distinguishing gross disease on imaging	Distinguishing normal vs. suspicious inguinal nodes.
Using additional available medical information efficiently	a. Endoscopy reports. b. Incorrectly neglecting endoscopy report (after neoadjuvant chemo). c. Need to use pre-chemo MRI.
Applying contouring tools, resources, and mental tricks	Educators demonstrate their style for how they contour and speak aloud their thoughts as they show contouring.
Contouring anatomic volumes and expansions	a. Make sure the CTV goes all the way down to the pelvic floor (not just doing a fixed superior and inferior expansion of the GTV). b. Make sure the anterior portion of the CTV goes all the way up to the bladder, even if the small bowel is between the rectum and bladder. Don’t skimp on the mesorectum. (Distinguish this vs. prostate/GYN). c. For T4 disease with anterior involvement, cover the external iliacs and cover the posterior bladder. d. A common mistake is covering the obturator nodes or inguinal nodes when they shouldn’t be in the CTV.

**Table 14:** Intact anus (Session 9) CTV: Clinical Target Volume; GTV: Gross Tumor Volume; DECREASE: De-Intensified ChemoRadiation for Early-Stage Anal Squamous Cell Carcinoma; OAR: Organ at Risk; IMRT: Intensity Modulated Radiotherapy; RTOG: Radiation Therapy Oncology Group

Section	Details
Pre-readings	- Anal cancer delineation of target volumes and critical structures DECREASE guide 2020 [52]. - Australasian anal cancer IMRT atlas 2011 [53]. - Anorectal contouring atlas for RTOG - Myerson et al. 2009 [51].
Distinguishing gross disease on imaging	Distinguishing normal vs. suspicious inguinal nodes.
Using additional available medical information efficiently	General tips.
Applying contouring tools, resources, and mental tricks	Educators demonstrate their style for how they contour and speak aloud their thoughts as they show contouring.
Contouring anatomic volumes and expansions	a. How to expand from GTVp to CTVp (p = primary disease). b. How to contour the inferior portion of the CTVp. c. OARs: genitalia, skin folds, sacral skin, bone marrow.

**Table 15:** Vulva (Session 10) CTV: Clinical Target Volume; GTV: Gross Tumor Volume

Section	Details
Pre-readings	- Consensus recommendations for radiation therapy contouring and treatment of vulvar carcinoma - Gaffney et al. 2016 [54].
Distinguishing gross disease on imaging	Distinguishing normal vs. suspicious inguinal nodes.
Using additional available medical information efficiently	General tips.
Applying contouring tools, resources, and mental tricks	Educators demonstrate their style for how they contour and speak aloud their thoughts as they show contouring.
Contouring anatomic volumes and expansions	a. How to expand from GTVp to CTVp (p = primary disease). b. Do you bridge the CTV between the inguinal nodes and the CTVp. c. Additional points per educator's discretion.

Supplement 3: Confidence and knowledge questions

Confidence Questions

The participants were asked to score the following confidence questions using a Likert scale (1: not confident, 2: a little confident, 3: somewhat confident, 4: quite confident, 5: extremely confident) [13].

Please indicate how confident you currently are in your ability to contour EVERY target volume without ANY errors for the following disease sites using the Likert scale: cervical cancer (external beam contouring), cervical cancer (brachytherapy contouring), endometrial cancer, intact prostate cancer, postoperative prostate cancer, bladder cancer (intact), rectal cancer (intact), rectal cancer (postoperative), anal cancer (intact), and vulvar cancer.

Please indicate how confident you currently are in your ability to perform the following using the Likert scale: 1. Contour the pelvic lymph node regions without ANY errors, accounting for differences in how they are drawn between disease sites. 2. Teach skills involved in contouring to a junior team member.

Knowledge Questions

Question 1:

Correct answer: Option d

Explanation: Elective lymph nodes should be contoured with a 0.7 cm uniform margin around the vessels (including arteries and veins). This can be done easily by using a 1.4 cm brush and keeping the center of the brush along the edge of the vessel as you contour. You may subtract bowel and bladder from the CTV.

Common mistakes: 

· Contouring only the vessel without any expansion (Option a)

· Applying a random/inconsistent margin around the vessels (Option c)

· Contouring with a 0.7 cm uniform margin, but not subtracting out any bowel or bladder from the CTV (Not shown)

Question 2:

Correct answer: Option b

Explanation: In rectal and anal cancers, there is a much higher chance of tumor recurrence occurring in the sacral hollow (where the sacral plexus nerve roots exit), compared to other pelvic cancers. Thus, this space should be contoured in high-risk CTV. The elective dose does not cause neuropathy, and it helps prevent recurrences that would otherwise be excruciatingly painful (on the nerves) and very difficult to salvage. 

The posterior aspect of the bladder (~1 cm) should be included in high-risk CTV slices in rectal and anal cancers to account for day-to-day potential variation in bladder filling and position.

Common mistakes: 

· Posterior part tries to “spare” the sacral nerve roots, not going back all the way against the anterior sacrum (Options a and d)

· Trying to carve out the bladder from the high-risk CTV (Options c and d)

Question 3:

For locally advanced cervical cancer, FIGO stage IIA, what is the correct way to extend the CTV into the vagina for external beam contouring?

a. The vagina should not be included in the CTV.

b. Cover the upper 2 cm vagina and, if involved, extend to 2 cm below the GTV.

c. Cover down to the introitus.

d. Cover the upper ½ vagina.

Correct answer: Option b

Explanation: In cases without gross vaginal involvement, cover the upper 2 cm of the vagina. If there is tumor extension into the vagina, then cover down to 2 cm below the GTV. Although some may say that covering down to the introitus can prevent recurrences, doing this routinely results in unnecessary severe vaginal toxicity and stricture. Brachytherapy is an excellent salvage option with good outcomes for local recurrence if detected early, and since this salvage option exists, only the upper portion of the vagina at the highest risk should be contoured.

Wrong answers: 

· Cover all the way to the introitus of the vagina. Unless the GTV extends very low and 2 cm below the GTV reaches the introitus, the CTV should not routinely extend this low (Option c)

· Just using a fixed distance of vagina for every case, regardless of the GTV (Option d)

· Not covering any of the vagina or too little of the upper ⅓ of the vagina (Option a)

Question 4:

For endometrial cancer, where would you define the upper border of the uterus/parametria surgical bed CTV?

a. Vaginal cuff, radio-opaque marker required for accurate identification.

b. Vaginal cuff plus any visible paravaginal/parametrial scar tissue, radio-opaque marker required for accurate identification.

c. Vaginal cuff plus any visible paravaginal/parametrial scar tissue, as seen on CT scan (without marker).

d. Vaginal cuff plus any visible paravaginal/parametrial scar tissue, CT (without marker) combined with MRI is sufficient for accurate identification.

Correct answer: Option b

Explanation: The upper border should be the vaginal cuff plus any visible paravaginal/parametrial scar tissue. This is extremely challenging to tell accurately on a CT scan alone. Therefore, a radio-opaque marker placed at the vaginal cuff at the time of CT simulation is required for accurate identification and CTV contouring. 

Not only does this help determine the upper border, but the lower border of the CTV can also be measured from this marker. eContour and the most recent NRG/Radiation Therapy Oncology Group (RTOG) 2021 guidelines recommend the vaginal lower border to be 3-4 cm from the vaginal cuff or 1 cm above the lower edge of the obturator foramen [12,22].

Wrong answers: 

· Option a: In addition to identifying the vaginal cuff, you should identify any visible paravaginal/parametrial scar tissue. The paravaginal/parametrial scar tissue should be included in the CTV and may require the upper border to be a little higher.

· Option c: As stated, it is extremely difficult and error-prone to rely just on a CT simulation scan without a marker.

· Option d: A postoperative MRI before contouring is helpful to determine the presence of paravaginal/parametrial scar tissue. However, it is still difficult to tell the border of the vaginal cuff with MRI alone. A radio-opaque marker should be placed at the time of CT simulation in addition to getting the MRI to sufficiently contour the upper extent of the CTV accurately.

Question 5:

Correct answer: Option d

Explanation: The apex of the prostate is where the levator ani muscles transition from a concave to a convex appearance at the GU diaphragm. This is easily seen on prostate MRI, but can be difficult to see in about 50% of CT Scans. There are ways to see if you are near the correct spot: if you are about 1.5 cm above the penile bulb or if you are near the inferior aspect of the pubic symphysis. However, these ways are not always accurate, and looking for the shape of the levator ani muscles to point out the GU diaphragm is the best way to identify the apex.

Common mistakes:

· Contouring the apex down too low, potentially missing the anatomic landmarks that distinguish it, and not recognizing the warning sign on the sagittal view that something doesn’t look like it was done correctly (Option a)

· Contouring the apex as a small contour that is repeated downward to make a stool shape. The apex is never shaped like this (Option b)

· Contouring the apex while staying attached to the rectum posteriorly (Option c). Rather, the prostate should have a natural, rounded shape on the sagittal view as the space between the rectum and apex naturally increases inferiorly.

Question 6:

Correct answer: Option a

Explanation: In the postoperative setting, the posterior part of the CTV should be touching the ENTIRE anterior surface of the rectal wall, including the lateral corners, making sure to cover that area comprehensively so that there aren’t recurrences anterior to the rectum. 

Common mistakes: 

· Posterior part tries to spare the rectum too much, not going back all the way against the anterior rectal wall (Options b, c, and d)

· Excluding the bladder (Option d)

Question 7:

Correct answer: Option b

Explanation: The sagittal view of the postoperative prostate CTV should have a “toilet seat” appearance, going up a little bit along the pubic symphysis anteriorly and then continuing up and including the posterior 1 cm of bladder up to the surgically cut vas deferens posteriorly (where the seminal vesicles existed preoperatively).

Common mistakes: 

· Mixing up the toilet seat appearance so that the anterior aspect goes higher than the posterior aspect (Options c and d)

· Not including the posterior 1 cm of the bladder in the postoperative CTV (Not shown)

· Not going up high enough for the posterior aspect of the postoperative CTV (Options c and d)

· Coming off the pubic symphysis too early as you go superiorly (Option a)

Question 8:

What is the standard of care approach for locally advanced bladder cancer when attempting bladder preservation?

a. 64 Gy in 32 fractions, to whole bladder and coverage for any tumor extension, plus a margin.

b. 55 Gy in 20 fractions to whole bladder and coverage for any tumor extension, plus a margin.

c. ≥61.2 Gy with conventional fractionation to the whole bladder, plus a lower dose to the prostatic urethra, plus a lower dose to the elective pelvic nodes, plus a margin.

d. There is no standard of care approach today.

Correct answer: Option b

Explanation: A meta-analysis of BC2001 (Bladder Cancer 2001) and BCON (Bladder Carbogen Nicotinamide) trials published in Lancet Oncology 2021 showed that hypofractionation with a uniform dose to the bladder (including any extra vesical tumor extension) plus a 1.5 cm PTV margin was non-inferior to conventional fractionation for toxicity and actually improved locoregional control [47].

Wrong answers:

· Option a is no better than hypofractionation and requires more resources.

· Option c is the RTOG protocol guideline, which has fallen out of favor now with the BC2001 findings plus the Lancet Oncology 2021 findings stated above.

· Option d is somewhat debatable, but we recommend taking a stance and strongly advocating for Option b as the new default standard.

Question 9:

Correct answer: Option a

Explanation: The CTV should maintain a margin around the rectum, even below the GTV. The inferior aspect of the CTV ends approximately 2 cm below the GTV. It does not extend down to the anal verge by default.

Common mistakes:

· Contouring the inferior aspect of the rectum down to the anal verge by default (Options b and d)

· Not including a margin around the rectum at the inferior aspect of the CTV (Options b and c)

Question 10:

Correct answer: Option c

Explanation: The obturator nodes should be covered along the pelvic sidewall. Additionally, a generous ≥2 cm margin should be included around the GTV. At this slice, it’s okay if that means it goes into the posterior bladder/prostate/seminal vesicles.

Common mistakes:

· Not including a wide axial margin around the GTV or rectum (Options a and b)

· Not including the obturator nodes sufficiently in the CTV for anal cancer (Options b and d)

## References

[1] Coleman CN, Formenti SC, Williams TR (2014). The international cancer expert corps: a unique approach for sustainable cancer care in low and lower-middle income countries. Front Oncol.

[2] Hatcher JB, Oladeru O, Chang B (2020). Impact of high-dose-rate brachytherapy training via telehealth in low- and middle-income countries. JCO Glob Oncol.

[3] Yorke AA, Carlson C, Koufigar S, Zhu H, Li B (2023). Impact of high-dose-rate brachytherapy training via telehealth in low- and middle-income countries. JCO Glob Oncol.

[4] Abrams RA, Winter KA, Regine WF (2012). Failure to adhere to protocol specified radiation therapy guidelines was associated with decreased survival in RTOG 9704 - a phase III trial of adjuvant chemotherapy and chemoradiotherapy for patients with resected adenocarcinoma of the pancreas. Int J Radiat Oncol Biol Phys.

[5] Loo SW, Martin WM, Smith P, Cherian S, Roques TW (2012). Interobserver variation in parotid gland delineation: a study of its impact on intensity-modulated radiotherapy solutions with a systematic review of the literature. Br J Radiol.

[6] Gillespie EF, Panjwani N, Golden DW (2017). Multi-institutional randomized trial testing the utility of an interactive three-dimensional contouring atlas among radiation oncology residents. Int J Radiat Oncol Biol Phys.

[7] Grimshaw JM, Russell IT (1993). Effect of clinical guidelines on medical practice: a systematic review of rigorous evaluations. Lancet.

[8] Kavuma A, Kibudde S, Schmidt M (2023). Remote global radiation oncology education and training: a pathway to increase access to high-quality radiation therapy services in low- and middle-income countries. Adv Radiat Oncol.

[9] Caicedo-Martínez M, Li B, González-Motta A, Carlson C, Zhu H, Bobadilla I, Martínez D (2023). Targeting education as a barrier to implement hypofractionation: results of a country-wide training program. Adv Radiat Oncol.

[10] Sarria GR, Timmerman R, Hermansen M (2022). Longitudinal remote SBRT/SRS training in Latin America: a prospective cohort study. Front Oncol.

[11] Hall WA, Paulson E, Davis BJ (2021). NRG Oncology updated international consensus atlas on pelvic lymph node volumes for intact and postoperative prostate cancer. Int J Radiat Oncol Biol Phys.

[12] Small W Jr, Bosch WR, Harkenrider MM (2021). NRG Oncology/RTOG consensus guidelines for delineation of clinical target volume for intensity modulated pelvic radiation therapy in postoperative treatment of endometrial and cervical cancer: an update. Int J Radiat Oncol Biol Phys.

[13] Likert R (1932). A technique for the measurement of attitudes. Arch Psych.

[14] Cacicedo J, Navarro-Martin A, Gonzalez-Larragan S, De Bari B, Salem A, Dahele M (2020). Systematic review of educational interventions to improve contouring in radiotherapy. Radiother Oncol.

[15] Mailhot Vega RB, De La Mata D, Amendola B (2021). Cross-sectional international survey to determine the educational interests of Spanish-speaking Latin American radiation oncologists. JCO Glob Oncol.

[16] Maitre P, Krishnatry R, Chopra S (2022). Modern radiotherapy technology: obstacles and opportunities to access in low- and middle-income countries. JCO Glob Oncol.

[17] Li B, Faúndez Salazar J, Rivera AF (2022). Radiation oncology residency training in Latin America: a call to attention. Adv Radiat Oncol.

[18] Ige T, Lewis P, Shelley C, Pistenmaa D, Coleman CN, Aggarwal A, Dosanjh M (2023). Understanding the challenges of delivering radiotherapy in low- and middle-income countries in Africa. J Cancer Policy.

[19] Li B, Perez T, Hao J, Rodriguez D, Oladeru O, Castaneda SA, Sarria GR (2022). Willingness to pay for high-quality remote radiation oncology training in Latin America. Crit Rev Oncol Hematol.

[20] Mitchell DM, Perry L, Smith S (2009). Assessing the effect of a contouring protocol on postprostatectomy radiotherapy clinical target volumes and interphysician variation. Int J Radiat Oncol Biol Phys.

[21] Fuller CD, Nijkamp J, Duppen JC (2011). Prospective randomized double-blind pilot study of site-specific consensus atlas implementation for rectal cancer target volume delineation in the cooperative group setting. Int J Radiat Oncol Biol Phys.

[22] (2025). eContour. https://www.econtour.org.

[23] (2025). RadOncReview - evidence, distilled. https://www.radoncreview.org.

[24] (2025). Radiopaedia, the peer-reviewed collaborative radiology resource. https://radiopaedia.org.

[25] (2025). e-Anatomy, the anatomy of imaging. https://www.imaios.com/en/e-anatomy.

[26] (2025). Rayos Contra Cancer training programs. https://www.rayoscontracancer.org/training-programs.

[27] De Hertogh O, Le Bihan G, Zilli T (2024). Consensus delineation guidelines for pelvic lymph node radiation therapy of prostate cancer: on behalf of the Francophone Group of Urological Radiation Therapy (GFRU). Int J Radiat Oncol Biol Phys.

[28] (2025). IMRT 2.0 clinical session 4, 6, & 8. Contouring concepts and pearls: GYN, peds, & prostate. https://www.youtube.com/watch?v=eo-gIbJx5Ck.

[29] (2025). Session 19 - live planning session #3 cervical cancer contouring. https://www.youtube.com/watch?v=rgpsfJslSZQ.

[30] (2025). Rayos Contra Cancer HDR brachytherapy live planning 3: cervical cancer contouring. https://www.youtube.com/watch?v=XrUjTPteEHo.

[31] Lim K, Small W Jr, Portelance L (2011). Consensus guidelines for delineation of clinical target volume for intensity-modulated pelvic radiotherapy for the definitive treatment of cervix cancer. Int J Radiat Oncol Biol Phys.

[32] Pötter R, Tanderup K, Kirisits C (2018). The EMBRACE II study: the outcome and prospect of two decades of evolution within the GEC-ESTRO GYN working group and the EMBRACE studies. Clin Transl Radiat Oncol.

[33] Eifel P, Klopp AH (2016). Gynecologic Radiation Oncology: A Practical Guide. https://scholar.google.com/scholar?q=intitle:Gynecologic%20radiation%20oncology%20%3A%20a%20practical%20guide.

[34] Lakomy DS, Wu J, Chapman BV (2022). Use of specific duodenal dose constraints during treatment planning reduces toxicity after definitive paraaortic radiation therapy for cervical cancer. Pract Radiat Oncol.

[35] (2025). Gynecologic cancer. https://www.nrgoncology.org/about-us/center-for-innovation-in-radiation-oncology/gynecologic-cancer.

[36] (2025). Intact prostate contouring guide. https://econtour.org/training/intact_prostate_module.pdf.

[37] Gay HA, Barthold HJ, O'Meara E (2012). Pelvic normal tissue contouring guidelines for radiation therapy: a Radiation Therapy Oncology Group consensus panel atlas. Int J Radiat Oncol Biol Phys.

[38] McLaughlin PW, Evans C, Feng M, Narayana V (2010). Radiographic and anatomic basis for prostate contouring errors and methods to improve prostate contouring accuracy. Int J Radiat Oncol Biol Phys.

[39] McLaughlin PW, Troyer S, Berri S, Narayana V, Meirowitz A, Roberson PL, Montie J (2005). Functional anatomy of the prostate: implications for treatment planning. Int J Radiat Oncol Biol Phys.

[40] Michalski JM, Lawton C, El Naqa I (2010). Development of RTOG consensus guidelines for the definition of the clinical target volume for postoperative conformal radiation therapy for prostate cancer. Int J Radiat Oncol Biol Phys.

[41] Thompson IM, Valicenti RK, Albertsen P (2013). Adjuvant and salvage radiotherapy after prostatectomy: AUA/ASTRO guideline. J Urol.

[42] Dal Pra A, Dirix P, Khoo V (2023). ESTRO ACROP guideline on prostate bed delineation for postoperative radiotherapy in prostate cancer. Clin Transl Radiat Oncol.

[43] (2026). ARRO-case postoperative radiotherapy in prostate cancer. https://www.astro.org/ASTRO/media/ASTRO/AffiliatePages/arro/PDFs/PostopProstate.pdf.

[44] Gunther JR, Liauw SL, Choi S (2016). A prostate fossa contouring instructional module: implementation and evaluation. J Am Coll Radiol.

[45] Baumann BC, Guzzo TJ, He J (2013). Bladder cancer patterns of pelvic failure: implications for adjuvant radiation therapy. Int J Radiat Oncol Biol Phys.

[46] Baumann BC, Bosch WR, Bahl A (2016). Development and validation of consensus contouring guidelines for adjuvant radiation therapy for bladder cancer after radical cystectomy. Int J Radiat Oncol Biol Phys.

[47] Choudhury A, Porta N, Hall E (2021). Hypofractionated radiotherapy in locally advanced bladder cancer: an individual patient data meta-analysis of the BC2001 and BCON trials. Lancet Oncol.

[48] Dinh T, Mitin T, Hoffman K (2020). Towards evidence based practice: the American Radium Society (ARS) and American College of Radiology (ACR) appropriate use guidelines on radiation therapy for muscle-invasive bladder cancer. Int J Radiat Oncol Biol Phys.

[49] James ND, Hussain SA, Hall E (2012). Radiotherapy with or without chemotherapy in muscle-invasive bladder cancer. N Engl J Med.

[50] Valentini V, Gambacorta MA, Barbaro B (2016). International consensus guidelines on clinical target volume delineation in rectal cancer. Radiother Oncol.

[51] Myerson RJ, Garofalo MC, El Naqa I (2009). Elective clinical target volumes for conformal therapy in anorectal cancer: a Radiation Therapy Oncology Group consensus panel contouring atlas. Int J Radiat Oncol Biol Phys.

[52] Damico N, Meyer J, Das P (2022). ECOG-ACRIN guideline for contouring and treatment of early stage anal cancer using IMRT/IGRT. Pract Radiat Oncol.

[53] Ng M, Leong T, Chander S (2012). Australasian Gastrointestinal Trials Group (AGITG) contouring atlas and planning guidelines for intensity-modulated radiotherapy in anal cancer. Int J Radiat Oncol Biol Phys.

[54] Gaffney DK, King B, Viswanathan AN (2016). Consensus recommendations for radiation therapy contouring and treatment of vulvar carcinoma. Int J Radiat Oncol Biol Phys.

